# Non-small Cell Lung Cancer with Squamous Features Metastatic to a Colonic Polyp: A Case Report of a Very Rare Presentation

**DOI:** 10.7759/cureus.4810

**Published:** 2019-06-03

**Authors:** Hazim S Bukamur, Rahoma Saad, Ibrahim Shahoub, Mark Gusack, Nancy J Munn

**Affiliations:** 1 Internal Medicine, Marshall University Joan C. Edwards School of Medicine, Huntington, USA; 2 Critical Care, University of Kentucky, Lexington, USA; 3 Pathology, MANX Enterprises, Ltd., Huntington, USA; 4 Pulmonary Medicine, Veterans Affairs Medical Center, Huntington, USA

**Keywords:** colonic polyp, non-small cell lung cancer, metastases

## Abstract

Among all malignant diseases, lung cancer is the most common on a worldwide basis. It is usually discovered incidentally on lung imaging studies or because of symptoms. The diagnosis is confirmed on biopsy material from the primary malignancy or from metastatic deposits. This is a report of metastatic lung cancer with squamous features discovered in an endoscopically removed colonic polyp. To our knowledge, there are only two prior reports of lung cancer being diagnosed in colonic polyps. We could not find any reports of lung cancer with squamous features metastatic to a colon polyp. In this case, the carcinoma was found in a polyp removed from a patient who presented with severe anemia and melena.

## Introduction

Close to half of all patients with lung cancer have been determined to have distant metastases at the time of death [1]. The most common extra-pulmonary sites of metastases include liver, brain, lymph nodes, and adrenal glands [1-3]. It is rare for metastatic deposits to arise in the gastrointestinal (GI) tract. It is even more rare to find metastatic disease in the colon [2,4] and extremely rare in a colon polyp. GI metastases represent a diagnostic challenge and are a sign of late-stage disease [1]. Multiple cases of GI metastases have been reported over the last few decades [5-9], but many more incidences have been found at autopsy [10,11] meaning that many go unnoticed to the patient’s detriment. The small bowel is the most common site for lung metastases to the GI tract with sporadic cases involving the large bowel, stomach, anus, sigmoid colon and cecum [1]. None have been reported for metastatic carcinoma with squamous features, perhaps, in part, due to the lack of molecular markers that can reliably determine the origin of squamous cell carcinomas.

## Case presentation

**Figure 1:**
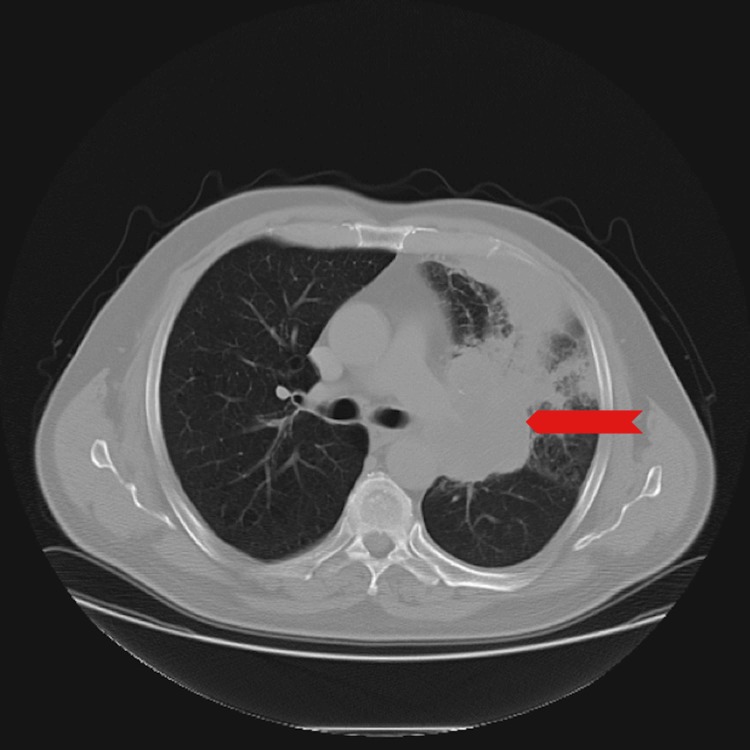
A CT chest showing left hilar mass.

A 59-year-old male presented with a three-week history of weakness and fatigue associated with black tarry stools. Past medical history was significant for smoking, essential hypertension, diabetes mellitus, coronary artery disease, and chronic obstructive pulmonary disease. Two years earlier, CT scan of the chest showed left hilar mass (Figure 1).

The patient was diagnosed with high-grade squamous cell carcinoma of the left upper lobe. At that time imaging studies showed metastatic disease to the brain. He was treated with palliative chemo-radiation therapy.

At this presentation, the patient was hypotensive with a blood pressure of 90/50 mm Hg, tachycardia at 110 beats per minute, and respiratory rate of 18 breaths per minute. He was afebrile. Physical examination revealed mild distress, moderate to severe dehydration, along with marked pallor of the skin and conjunctivae. His cardiopulmonary, abdominal, and neurological examination was unremarkable.

The laboratory workup revealed marked anemia with hemoglobin of 3.9 g/dl. All other laboratory tests were within reference ranges, including white blood cell count, platelet count, liver function panel, a metabolic panel, and PT/INR. CT scan of the pelvis and abdomen was negative for acute abnormalities or masses. He was admitted to the medical intensive care unit (ICU), started on intravenous (IV) fluid resuscitation, and transfused with four units of packed red blood cells.

**Figure 2:**
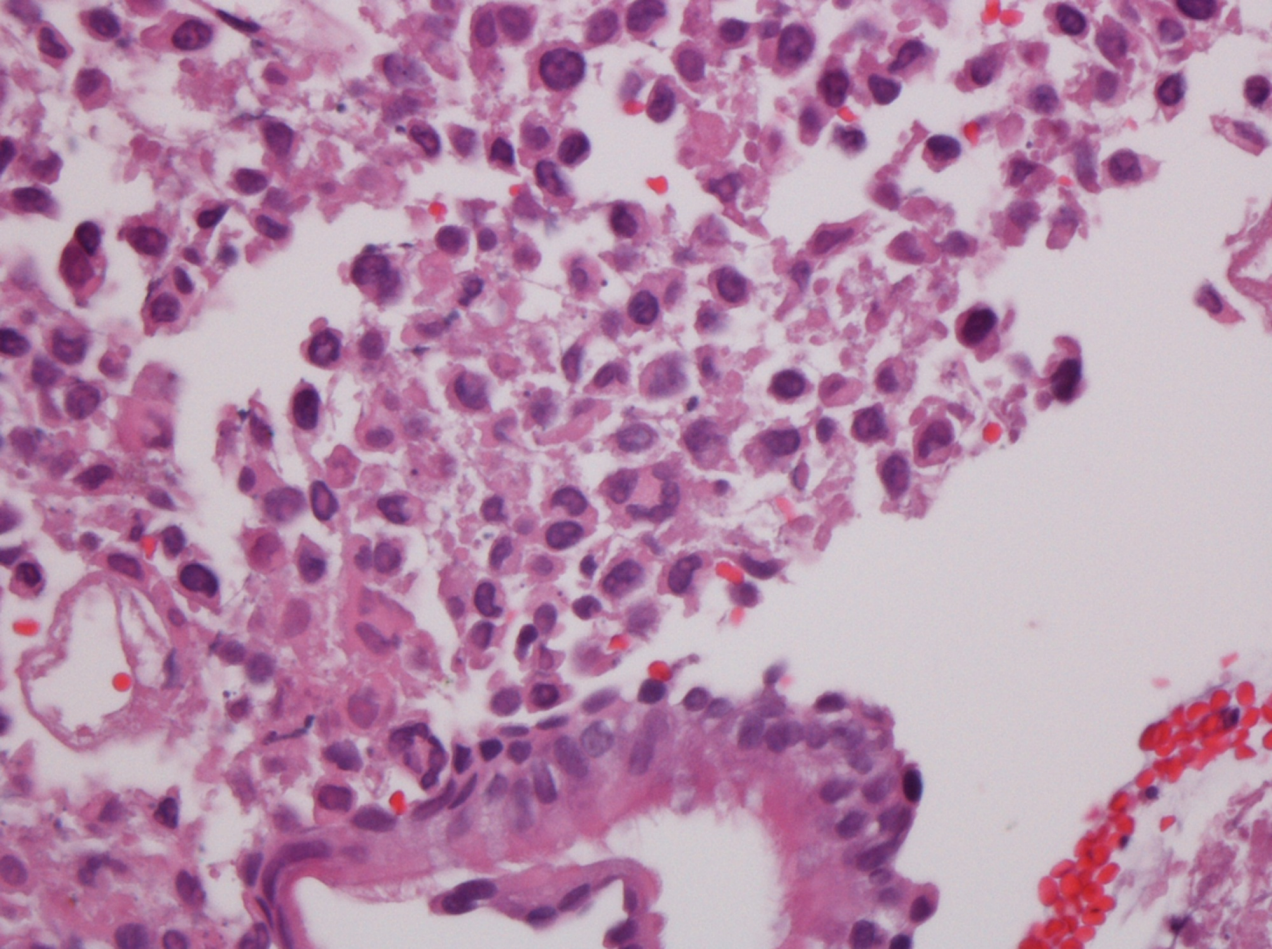
Lung mass: haematoxylin and eosin stain 40x, non-small cell carcinoma, poorly differentiated, located beneath the normal bronchial mucosa.

**Figure 3:**
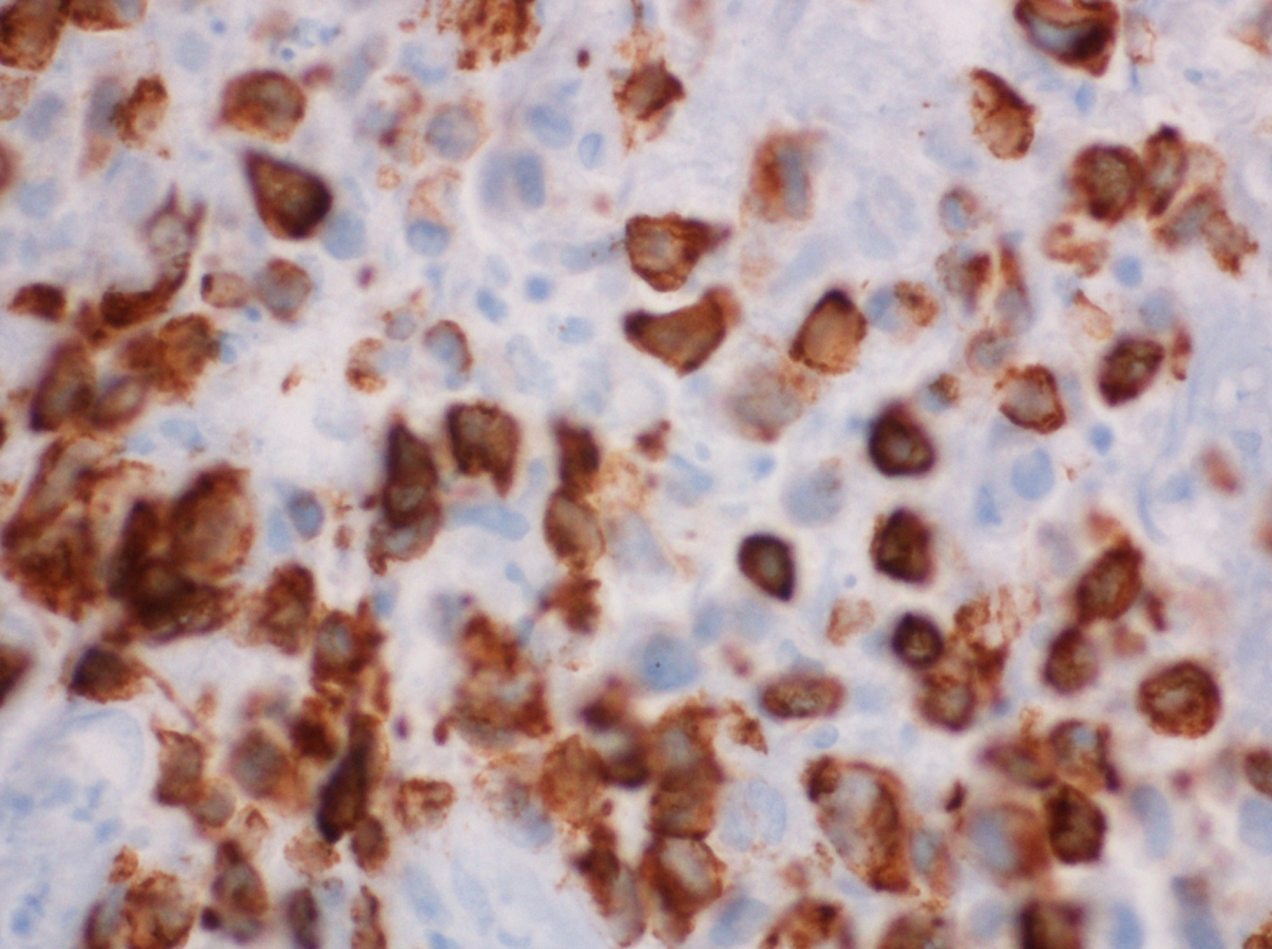
Lung mass: immunohistochemical stain 60x, many cells are cytokeratin 7 (CK7) positive.

**Figure 4:**
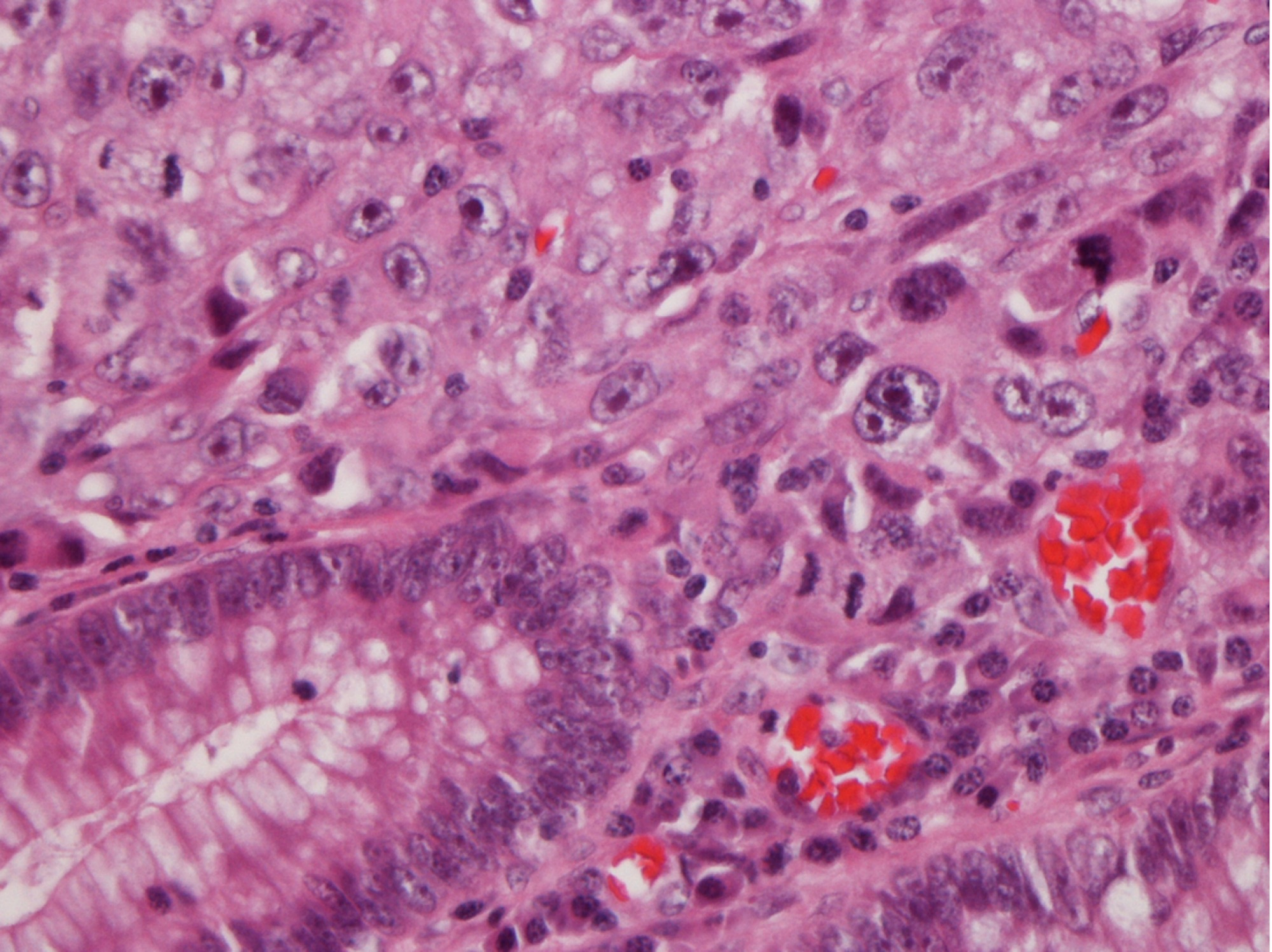
Colon polyp: haematoxylin and eosin stain 40x, non-small cell carcinoma, poorly differentiated, undermining mucosa with the same morphology.

**Figure 5:**
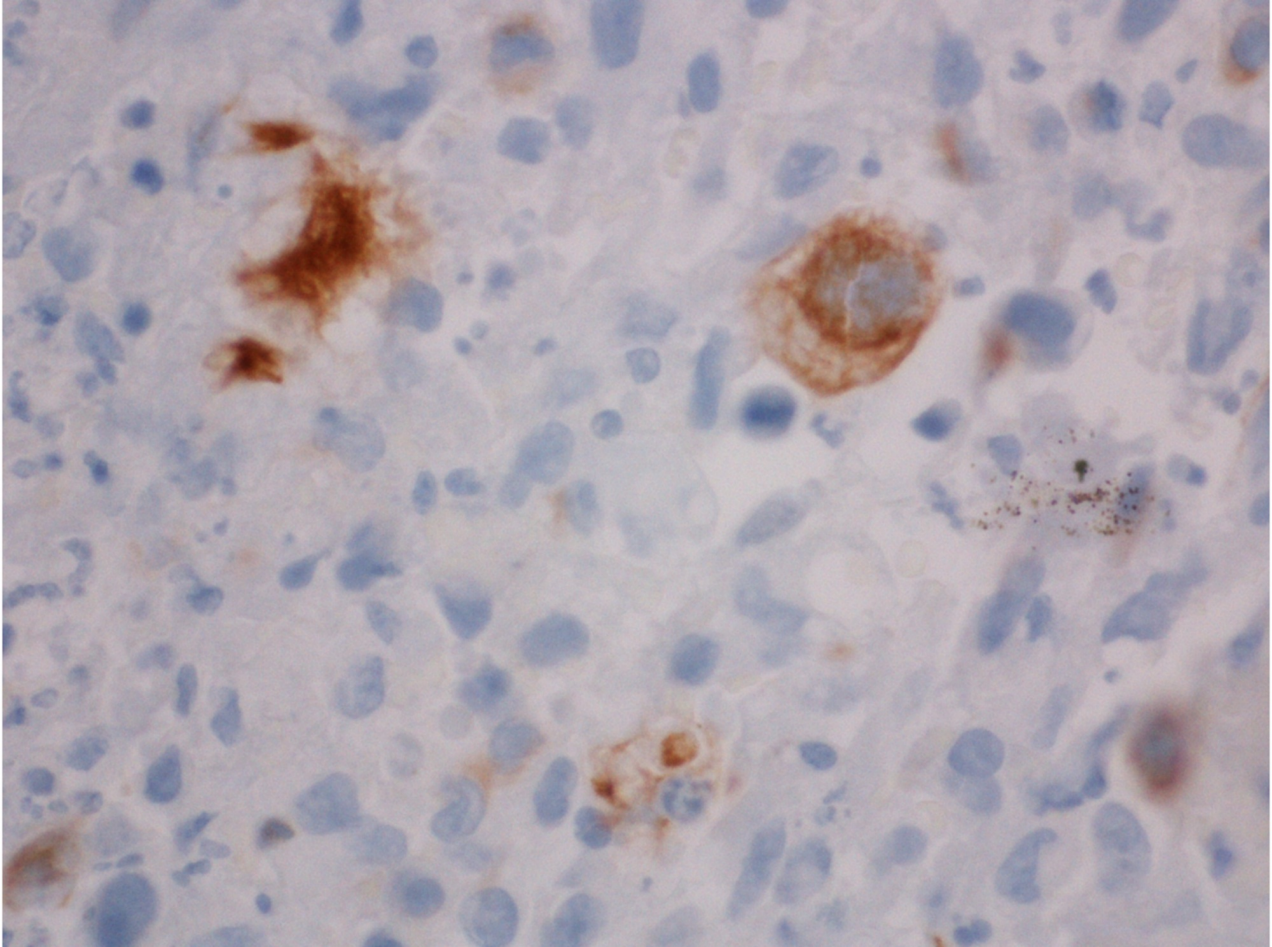
Colon polyp: immunohistochemical stain 60x, cytokeratin 7 (CK7) positive. Not as many cells are positive but the full immunohistochemical analysis is the same.

After the patient was stabilized, colonoscopy was carried out revealing multiple colonic polyps which were removed and sent to the laboratory for diagnostic evaluation. One polyp showed sheets of poorly differentiated malignant cells subjacent to the overlying mucosa. These were positive for pan-keratin and negative for S100, HMB 45, CD20, CD45RO, chromogranin and synaptophysin (Figures 2-5).

The histologic morphology matched the tumor identified two years earlier in the left upper lobe of the lung. Of note, the patient had upper and lower endoscopy two years prior this presentation. At that time, the upper GI tract appeared normal while two benign hyperplastic polyps were removed from the lower GI tract.

## Discussion

Polyps harboring metastasis from extra-colonic can­cers have been reported in patients with breast, ovarian, skin (melanoma), gastric, esophageal and renal cancer [2,12]. From our review of published cases of lung cancer metastatic to the GI tract only two report metastatic deposits in colonic polyps [2,4]. Reports of lung carcinoma with squamous features metastatic to colonic polyps were not found.

Both reported the metastatic tumor as non-small cell lung cancer. One characterized the tumor as adenocarcinoma(4) whereas the other report did not specify the histologic type [2]. Both of those cases were asymptomatic and found incidentally. One of the patients died of his malignancy [4] and the other one died of a pulmonary embolism [2]. In contrast to these two cases, our patient was symptomatic presenting with hypotension and lower GI bleeding leading to severe anemia that required a blood transfusion. This presentation led to further evaluation which revealed colonic polyps, one of which harbored the high-grade squamous cell carcinoma.

Gastrointestinal metastases usually have severe symptoms that adversely impact the quality of the patient’s life, for example, GI hemorrhage, abdominal discomfort, and even intestinal obstruction [1]. These metastases may also cause other severe life-threatening situations such as perforation of the bowel [13]. Unfortunately, it can be difficult to differentiate metastatic lung disease from primary GI malignancy based on imaging studies and endoscopic findings alone. It requires pathologic confirmation on biopsy material. Even then, unless the tumor retains some degree of differentiation, it may be impossible to confirm that the source is from a lung primary [14].

Therefore, it is necessary to utilize immunohistochemical staining on the biopsy material to attempt to determine if the tumor originated in the colon or is a metastasis from the lung while ruling out a third source such as breast, ovarian, skin (melanoma), gastric, esophageal and renal cancers [2]. The absence of reports of metastatic lung cancer (squamous cell carcinoma) to colonic polyp in the literature may be due to both rarity of this event combined with the lack of molecular markers that can reliably determine the origin of this type of tumor.

Depending on the extent and severity of the disease as well as presentation, the modalities of treatment can vary [15]. Chemotherapy is the main strategy utilized for patients with metastatic disease to distant organs. However, because chemotherapy increases the risk of a GI bleed and even perforation, most physicians might hesitate to offer this in these patients. Surgical resection for symptomatic relief should be considered in those patients with life-threatening conditions. In particular, for patients with isolated GI metastases this may also impact long-term survival [15].

## Conclusions

Our report represents a very rare case of primary lung cancer metastatic to a colonic polyp. This raises the issue that patients with advanced primary lung cancer and gastrointestinal symptoms may harbor metastases to the GI tract and even to a colonic polyp. Timely workup and evaluation may be of value for early detection and treatment in order to prevent serious complications and to potentially improve the patient’s outcome.
